# A real-time and high-throughput neutralization test based on SARS-CoV-2 pseudovirus containing monomeric infrared fluorescent protein as reporter

**DOI:** 10.1080/22221751.2021.1925163

**Published:** 2021-05-18

**Authors:** Wen-Yang Tsai, Lauren L. Ching, Szu-Chia Hsieh, Marian E. Melish, Vivek R. Nerurkar, Wei-Kung Wang

**Affiliations:** a Department of Tropical Medicine, Medical Microbiology and Pharmacology, John A. Burns School of Medicine, University of Hawaii at Manoa, Honolulu, HI, USA; b Pacific Center for Emerging Infectious Diseases, John A. Burns School of Medicine, University of Hawaii at Manoa, Honolulu, HI, USA; c Kapi’olani Medical Center for Women and Children, Honolulu, HI, USA; d Department of Pediatrics, John A. Burns School of Medicine, Honolulu, HI, USA

**Keywords:** SARS-CoV-2, pseudovirus, reporter, monomeric infrared fluorescent protein, neutralization

## Abstract

Neutralizing antibodies to SARS-CoV-2 have been shown to correlate with protection in animals and humans, disease severity, survival, and vaccine efficacy. With the ongoing large-scale vaccination in different countries and continuous surge of new variants of global concerns, a convenient, cost-effective and high-throughput neutralization test is urgently needed. Conventional SARS-CoV-2 neutralization test is tedious, time-consuming and requires a biosafety level 3 laboratory. Despite recent reports of neutralizations using different pseudoviruses with a luciferase or green fluorescent protein reporter, the laborious steps, inter-assay variability or high background limit their high-throughput potential. In this study we generated lentivirus-based pseudoviruses containing a monomeric infrared fluorescent protein reporter to develop neutralization assays. Similar tropism, infection kinetics and mechanism of entry through receptor-mediated endocytosis were found in the three pseudoviruses generated. Compared with pseudovirus D614, pseudovirus with D614G mutation had decreased shedding and higher density of S1 protein present on particles. The 50% neutralization titers to pseudoviruses D614 or D614G correlated with the plaque reduction neutralization titers to live SARS-CoV-2. The turn-around time of 48–72 h, minimal autofluorescence, one-step image quantification, expandable to 384-well, sequential readouts and dual quantifications by flow cytometry support its high-throughput and versatile applications at a non-reference and biosafety level 2 laboratory, in particular for assessing the neutralization sensitivity of new variants by sera from natural infection or different vaccinations during our fight against the pandemic.

## Introduction

The pandemic of coronavirus disease 2019 (COVID-19), first recognized in Wuhan, China in late 2019, is caused by the severe acute respiratory syndrome coronavirus 2 (SARS-CoV-2) [1,2]. Within the Coronaviridae family, there are four low pathogenic human coronaviruses (229E, NL63, OC43 and HKU1) causing mild to moderate respiratory illness in healthy individuals and three highly pathogenic coronaviruses (SARS-CoV-1, MERS-CoV and SARS-CoV-2) causing pneumonia, respiratory distress and/or death [3,4].

As the major target of neutralizing antibodies, the spike (S) protein of SARS-CoV-2 contains 1273 amino acids and is cleaved by furin proteases along the secretory pathway into S1, which contains a receptor binding domain (RBD) and binds to ACE2 receptor, and S2 subunits [3–6]. The S2 subunit is further cleaved by host cell serine protease TMRPSS2 to S2’ subunit which is involved in the fusion step of entry [3,4,7]. At the S1-S2 junction, SARS-CoV-2 contains a multibasic cleavage site (RRAR) which has been shown to promote syncytial formation and infection [8–10]. A variant SARS-CoV-2 containing a D614G substitution in the S1 subunit and three other associated mutations first reported in Europe has become the dominant strain globally [11].

Working with live SARS-CoV-2 requires a biosafety level 3 laboratory and is labour intensive and time-consuming [12]. Several types of pseudoviruses expressing the S protein and a reporter in the backbone of an envelope gene-defective viral vector such as vesicular stomatitis virus (VSV), retrovirus and lentivirus that can complete one-round replication in target cells have been developed to study cellular tropism, neutralizing antibodies, mechanism of entry and entry inhibitors in a biosafety level 2 (BSL2) laboratory [13–16].

Commonly used reporters including various luciferases and green fluorescent proteins (GFPs) have several limitations. For cell-based luciferase assays, multiple and time-consuming steps including gentle aspiration of culture media, adding accurate amounts of lysis buffer for complete cell lysis and/or homogenization, adding accurate amounts of substrates and transfer to a new plate for reading together with the inter-assay variability limit the potential for high throughput. The relative high cost of substrates further makes luciferase assay undesirable. Although live-cell luciferase assays do not require cell lysis, intracellular ATP levels and uneven distribution of permeable luciferin may increase variability [17,18]. On the other hand, GFP-based assays had high background signals caused by autofluorescence from intracellular NADPH, flavin or extracellular collagen [19,20]. Several bright and stable near infra-red fluorescent proteins have been developed with a major advantage of lower autofluorescence and lighter scattering compared with GFP [21,22], and used as a real-time marker to monitor the growth of cancer cells [18] and replication of recombinant virus in vivo [23]. The recently reported monomeric infrared fluorescent protein (miRFP) further advanced its applications for in vivo imaging, protein–protein interaction, and reporter assays [24].

Recent studies have shown that neutralizing antibodies against SARS-CoV-2 correlate with protection in non-human primates and humans, disease severity, survival, and vaccine efficacy [25–28]. With current large-scale immunizations and vaccine trials in different countries together with the emergence and spread of new variants [11,29–31], there is a critical need for a simple, cost-effective and high-throughput neutralization test. In this study, we developed lentivirus-based SARS-CoV-2 pseudoviruses containing miRFP as a real-time reporter for high-throughput neutralization and have tremendous applications to our fight against the pandemic.

## Materials and methods

### Human subjects

The study of coded serum samples has been approved by Institutional Review Boards (IRB) of the University of Hawaii (2020-00406). After informed consent was obtained, the samples were collected in Honolulu, Hawaii between July and October, 2020 including sixteen convalescent-phase samples (16-188 days post-symptom onset) from 15 reverse-transcription polymerase-chain reaction (RT–PCR)-confirmed COVID-19 cases and one with positive COVID-19 antibody test as well as two samples from individuals who had contacts with COVID-19 cases but RT–PCR negative.

### Plasmids

The miRFP gene franked by NotI and XhoI sites was synthesized and codon-optimized (Integrated DNA Technologies) [24], digested with restriction enzyme and cloned into an *env*-defective HIV-1 reporter construct pNL4-3.Luc.R-E- to generate pNL4-3 R-E-miRFP [32] (AIDS Reagent Bank). Plasmid pCAGGS expressing the S protein of SARS-CoV-2 Wuhan-Hu-1 strain was from BEI Resources. A plasmid expressing S protein with truncation of the C-terminal 19 amino acids, designated as Str, was generated by PCR amplification of pCASGGS and cloning into an expression vector pCB [33]. Plasmids expressing S proteins with D614G mutation and furin cleavage site mutation (RRAR→AAAR), designated as D614G and AAAR, respectively, were generated by site-directed mutagenesis of the plasmid Str. Plasmid expressing VSV glycoprotein (pVSV-G) has been described previously [34].

### Cells

HEK-293 T, HEK-293T-hACE2, Vero, Vero-E6 and Huh-7 cells were maintained in DMEM (Gibco) supplied with 10% FBS, 2% HEPES and 1% Penicillin–Streptomycin.

### Production of SARS-CoV-2 pseudoviruses

To optimize the pseudovirus production, HEK-293T cells were seeded in 6-well plate one day before transfection to reach 90–95% confluence, co-transfected with 2 µg pNL4-3 R-E-miRFP and 0.5, 1, 2, or 4 µg of plasmid Str using lipofectamine 2000, and incubated with 2 mL DMEM media containing 10% FBS. The supernatants were collected at 48 h post transfection, followed by low-speed centrifugation at 300× g for 10 min, passage through 0.45 μm syringe filter, aliquot and store at −80°C. To generate pseudoviruses for infection and neutralization, HEK-293T cells were seeded in 10-cm dish one day before transfection and co-transfected with 12 µg pNL4-3 R-E-miRFP and 3 µg of plasmids Str, D614G, AAAR or pVSV-G. To quantify viral genome copies in pseudoviruses, viral RNA in supernatants was extracted by QIAamp viral RNA mini kit (Qiagen) and quantified by qRT-PCR using iTaq one-step RT-qPCR kit (Bio-Rad) and primers targeting HIV-1 pol gene [35] using Applied Biosystems 7500 together with known amounts of pNL4-3 R-E-miRFP for standard curves [16,36].

### Pseudovirus infection

Cells (2 × 10^4^ cells/well) were seeded onto 96-well plates (Greiner Bio-One) one day before infection. After adding 150 µL (or serial 3-fold dilutions) of pseudoviruses per well, the plates were incubated at 37°C for 1 h (regular infection) or centrifugated at 1,200× g at 4°C for 1 h and incubated at 37°C for 1 h (spin infection) [14,37]. To test the effect of polybrene on pseudovirus infection, polybrene was added to each well (final 5 µg/mL) before spin infection. After adding DMEM media with 10% FBS, the plates were incubated at 37°C, 5% CO2 and scanned from 24 to 120 h post-infection using Li-Cor Odyssey CLx near-infrared fluorescence imaging system (at 700 nm) and image studio software 4.0. For infection kinetics in different cells, 1.65 × 10^9^ RNA copies of pseudovirus (∼75 µl of Str) per well were inoculated. For flow cytometry, the plate was washed with 1× PBS once followed by trypsinization, washing with 1× PBS with 2% FBS, fixation with 2% paraformaldehyde on ice for 15 min and washing. The percentage of positive cells were counted using Attune NxT flow cytometer and FlowJo software.

### Pseudovirus neutralization test

HEK-293T-hACE cells (2 × 10^4^ cells/well) were seeded onto 96-well plates one day prior to infection. Pseudovirus Str or D614G was mixed with 4-fold serial dilutions of serum at 1:1 ratio (75 µL/75 µL), incubated at 37°C for 1 h, and added to each well for spin infection. At 72 h, the plates were scanned by Li-Cor Odyssey imager. The % of infection at different serum dilutions were calculated by the formula (intensity of serum + pseudovirus – intensity of media only)/(intensity of pseudovirus only – intensity of media only) × 100. The % neutralization=100 – % of infection [13]. NT_50_ titer was the serum dilution that reached 50% neutralization using 4-parameter nonlinear regression analysis (GraphPad 6.0) [15,38].

### NH4Cl treatment

HEK-293T-hACE cells (2 × 10^4^ cells/well) were seeded onto 96-well plates and pretreated with 100 µL of serial 2-fold dilutions of NH4Cl (25–1.6 mM) at 37°C for 1 h, followed by spin infection with pseudoviruses prepared in fresh media containing NH4Cl (25–1.6 mM) [14,37]. The plate was incubated at 37°C for 18 h or overnight before replacing with fresh media and scanned at 48 h by Li-Cor Odyssey imager. The IC_50_ was the concentration of NH4Cl that inhibited 50% of pseudovirus infection compared to untreated control using 4-parameter nonlinear regression (GraphPad 6.0).

### SARS-CoV-2 PRNT

This assay was performed as previously described [39]. Briefly, Vero E6 cells (ATCC CRL-1586) were grown in 6-well plate seeded at 2 × 10^5^ cell/well in 3 mL 1× DMEM (2% HEPES, 1% penicillin–streptomycin) with 10% FBS three days earlier, and incubated at 37°C with 5% CO_2_ to achieve 100% confluence. On the day of assay each sample was serially diluted (1:10, 1:40, further serial 2-fold) in 1× DMEM with 2% FBS, and incubated with an equal volume of 50–100 plaque forming units of SARS-CoV-2 isolate USA-WA1/2020 (BEI Resources) at 37°C for 30 min. Vero E6 cells were inoculated with virus/serum mixture, incubated at 37°C with 5% CO_2_ for 1 h, and immobilized with a 2% agar overlay prepared with 2× DMEM with 4% FBS. Two days post-infection, a second overlay containing 0.33% Neutral red was added to visualize plaque formation, which were recorded after 12–24 h of additional incubation. Sigmoidal dose–response with variable slope simple logistical regression model was used to determine titers at 50% (PRNT_50_), 80% (PRNT_80_), and 90% (PRNT_90_) neutralization (GraphPad 9.0).

### Western blot analysis

Pseudoviruses Str, D614G and AAAR were concentrated by 20% sucrose cushion ultracentrifugation at 110,000× g and 4°C for 5 h and resuspended in 1× PBS [16,36,38]. After adding non-reducing sample buffer and boiling at 95°C for 2 min, the samples were subjected to 12% polyacrylamide gel electrophoresis, followed by transfer to nitrocellulose membrane, hybridization with rabbit sera and a human mAb CR3022 against SARS-CoV-1 S protein and RBD, respectively (BEI Resources), pooled HIV-1 positive sera [40], and secondary antibodies (IRDye^®^ 680RD-conjugated goat anti-rabbit IgG at 1:5000; IRDye^®^ 800CW-conjugated goat anti-human IgG at 1:10000). The signal was detected by Li-Cor Odyssey classic imager (LiCor Biosciences) with Image Studio software to quantify S1, S2 and S proteins.

### IgG binding by microsphere immunoassay

Anti-SARS-CoV-2 S1 and nucleocapsid (N) IgG antibodies were evaluated by a laboratory developed multiplex microsphere immunoassay as descried previously [41]. Briefly, magnetic carboxylated microspheres (MagPlexTM-C, Luminex) were coupled to recombinant SARS-CoV-2 S1 (SinoBiological) and N (Native Antigen Company) proteins, bovine serum albumin (BSA), or PBS [41]. Coupled microspheres were combined and diluted in 1× PBS-1% BSA at a dilution of 1/200. Then 50 μL (containing ∼1250 beads of each type) of the microsphere suspension and 50 μL of diluted serum (1:400) were added to each well of a black flat-bottom 96-well plate in duplicate and incubated on a plate shaker set at 700 rpm in the dark at room temperature for 3 h. After washing twice with 200 μL of PBS-BSA using a magnetic plate separator, 50 μL of red phycoerythrin-conjugated *F*(ab’)2 fragment goat anti-human IgG specific to the Fc*γ* fragment (Jackson Immunoresearch) diluted to 1 μg/mL were added to each well, and incubated for 45 min. After washing twice as above, microspheres were resuspended in 100 μL of sheath fluid and analysed on the Luminex 200 (Luminex). Data acquisition detecting the median fluorescence intensity (MFI) was set to 50 beads per spectral region in 100 µL, and double discriminator gating set at 7,500–19,000. Antigen-coupled beads were recognized and quantified based on their spectral signature and signal intensity, respectively. Assay cutoff values were determined based on the mean MFI value of 157 SARS-CoV-2 naïve samples (collected prior to the COVID-19 pandemic and/or SARS-CoV-2 PRNT negative) plus 3 standard deviations, which gave a confidence level higher than 99.9%. Test serum samples with MFI values greater than the cutoff were considered positive. Control microspheres were coupled to BSA and PBS to check for nonspecific attachment of serum proteins to the microspheres and signals generated for these microspheres were below cutoff values for all sera evaluated in this study. Samples were evaluated for anti-SARS-CoV-2 RBD IgG using the xMAP SARS-CoV-2 Multi-Antigen IgG assay (Luminex) per manufacture’s instruction.

### Statistical analysis

The two-tailed Mann–Whitney test and two-way ANONA were used to compare miRFP intensity between two groups and multiple groups, respectively (GraphPad 6.0). The two-tailed Spearman correlation test was used to determine the relationship between pseudovirus NT_50_ titers and SARS-CoV-2 PRNT_50_ titers or the sampling time post symptom onset; the two-tailed Wilcoxon rank signed test the NT_50_ titers to pseudoviruses Str and D614G (GraphPad 6.0).

## Results

### Generation of SARS-CoV-2 pseudovirus with miRFP reporter

**Figure 1.**
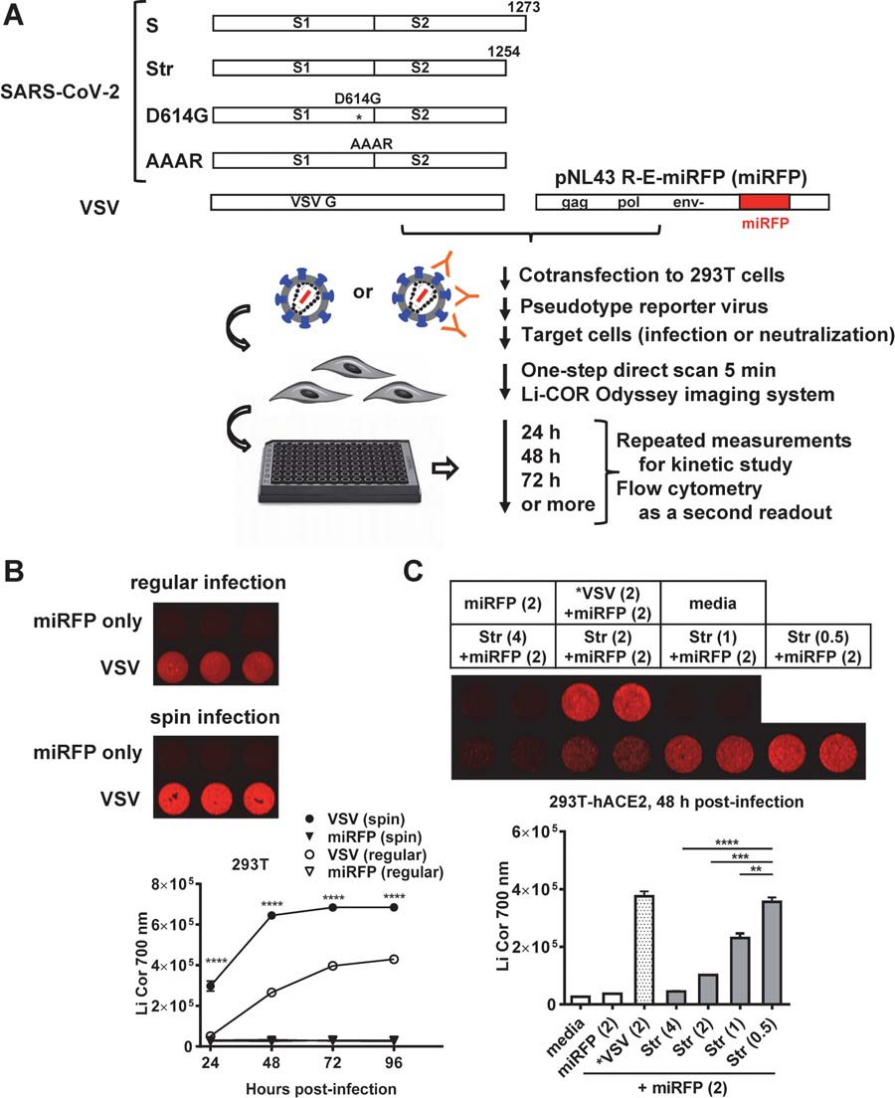
Generation of SARS-CoV-2 pseudoviruses containing miRFP reporter. (A) Schematic drawing of plasmids expressing full-length (S) and truncated (Str) SARS-CoV-2 S proteins, truncated S proteins with mutations (D614G and AAAR), and VSV-G protein as well as co-transfection with pNL43 R-E-miRFP (miRFP) to generate different pseudoviruses containing miRFP reporter, infection of target cells, one-step imagining and various applications. (B) Regular and spin infections of VSV pseudovirus to HEK-293T-hACE2 cells with miRFP signals at 48 h post-infection (top) and infection kinectics at 24–96 h post-infection (bottom). Data are means and standard deviations of triplicates from one representative experiment of two. (C) Yields of SARS-CoV-2 pseudovirus Str determined in HEK-293T-hACE2 cells at 48 h post-infection comparing different ratios of plasmid to vector (4, 2, 1, 0.5 µg/2 µg) during transfection, media, miRFP vector only and VSV pseudovirus (2 µg/2 µg) as positive control. miRFP signals (top) and quantification (bottom). **** *P* < 0.0001, *** *P* < 0.001, ** *P* < 0.01 by two-way (B) and one-way (C) ANONA (GraphPad 6.0).

We constructed a lentivirus vector containing miRFP, designated as pNL43 R-E-miRFP. In addition to pCAGGS, designated as plasmid S, which expresses the S protein of SARS-CoV-2 Wuhan-Hu-1 strain (containing D614), we generated 3 plasmids to facilitate the incorporation of S protein into pseudovirus: plasmid Str expressing S protein with truncation of C-terminal 19 amino acids, an ER-Golgi retention signal, plasmid D614G containing the D614G substitution and plasmid AAAR containing the AAAR mutation at the multibasic cleavage site, both in the Str backbone (Figure 1(A)). After generating different pseudoviruses through co-transfection, we first tested the infectivity of VSV-G pseudovirus by two methods. Whereas background signal was detected in the vector control, spin infection resulted in miRFP signals significantly higher than regular infection from 24 to 96 h post-infection (Figure 1(B)); spin infection was thus used for this study. Since the infectivity of pseudovirus Str containing truncated S protein was much higher than that containing wild type S protein (Figure S1(A)), we focused on the pseudovirus Str to further test the yields with different ratios of plasmid to vector. In HEK-293T-hACE2 cells, the yield of Str pseudovirus, though lower than VSV-G pseudovirus at a ratio of 2/2 µg, increased 7.9-fold as the ratio decreased from 4/2 µg to 0.5/2 µg (Figure 1(C)), supporting the generation of high-yield SARS-CoV-2 pseudovirus by a ratio of 0.5/2 µg in transfection. Based on higher yields of day 2- than day 3-collected pseudoviruses and no increase in infectivity by polybrene (Figures S1(B) and S1(C)), day 2-collected pseudoviruses without polybrene were used for further experiments.

### Infection kinetics and tropism of SARS-CoV-2 pseudoviruses with miRFP reporter

**Figure 2.**
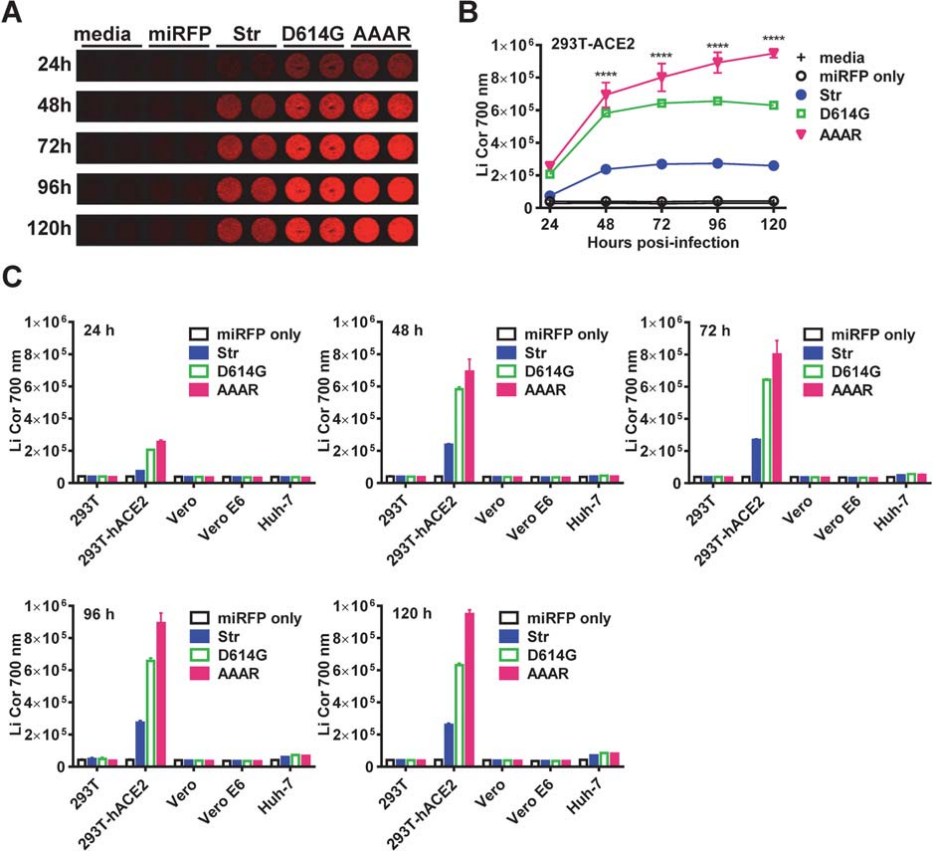
Infection kinetics and tropism of SARS-CoV-2 pseudoviruses with miRFP reporter. Infection kinectics of three SARS-CoV-2 pseudoviruses (Str, D614G, AAAR) in HEK-293T-hACE2 cells with miRFP signals (A) and quantification (B), and cellular tropism in different cells with miRFP quantification from 24 h to 120 h post-infection (C). Data are means and standard deviations of duplicates from 2 experiments.

We next examined the infection kinetics of 3 pseudoviruses (Str, D614G and AAAR) in different target cells. In agreement with previous reports [11,14,15], the highest signals were found in HEK-293T-hACE2 cells followed by Huh-7 (after 72 h) and background signals in other cells (Figures 2(A) and 2(C)), suggesting that SARS-CoV-2 pseudoviruses can infect HEK-293T-hACE2 cells efficiently and Huh-7 cells, though weakly, but not other cells tested. The infectivity of pseudovirus AAAR was higher than that of D614G, which in turn was higher than that of Str (Figures 2(A)-2(C)); the peak signals were between 72 and 96 h post-infection for Str and D614G and >120 h for AAAR probably due to its ability of syncytial formation (Figure 2(B)) [9].

### Characterization of SARS-CoV-2 pseudoviruses with miRFP reporter

**Figure 3.**
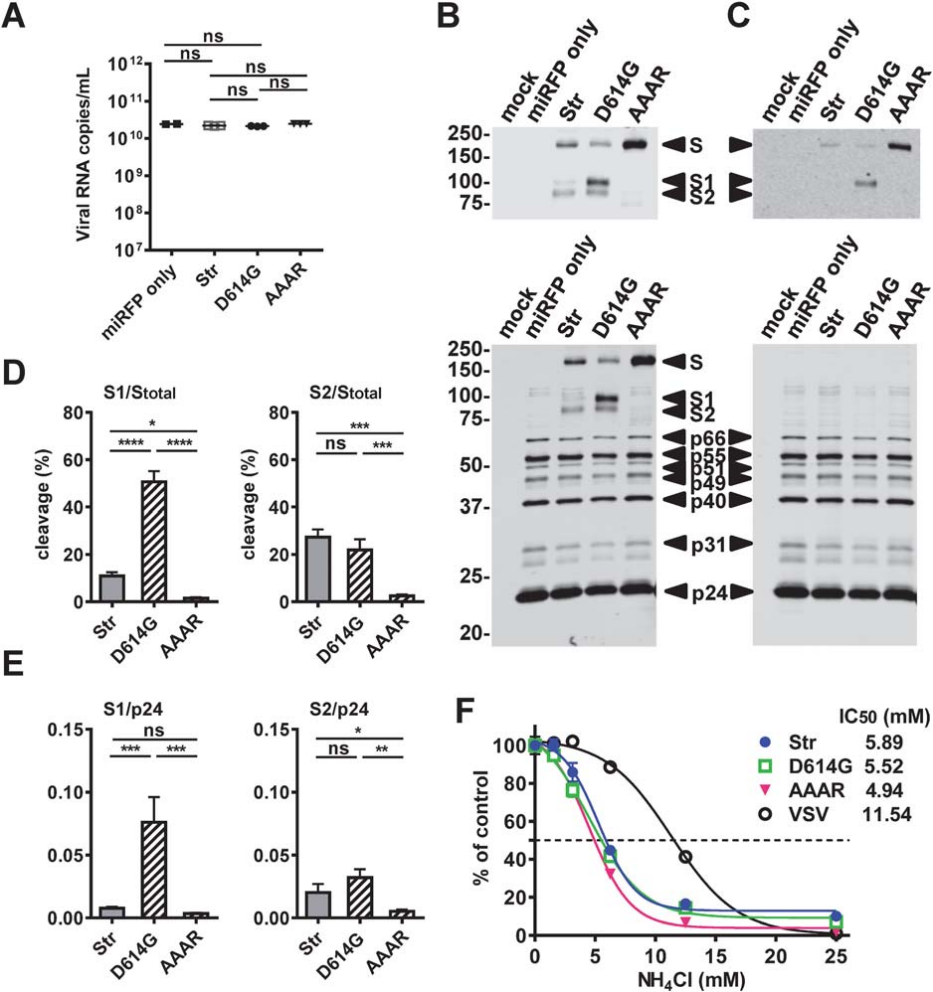
Characterization of SARS-CoV-2 pseudoviruses with miRFP reporter. (A) Quantification of viral RNA copies of three pseudoviruses (Str, D614G, AAAR) and miRFP vector only by qRT-PCR with primers targeting the HIV-1 pol gene. Data are means and standard deviations of 3 experiments. ns, not significant by Mann-Whitney test (GraphPad 6.0). (B,C) Western blot analysis of three pseudovirus particles (Str, D614G, AAAR) and miRFP vector only purified from sucrose cushion ultracentrifugation using HIV-1 positive sera (B,C bottom) and rabbit sera to S protein (B top) and a human mAb CR3022 to RBD (C top) of SARS-CoV-1. Gels are from one representative experiment of three. (D,E) Percentage of cleavage based on the ratio of the intensity of S2 or S1 protein band to that of total S protein (D) and the ratio of the intensity of S2 or S1 protein to p24 (E) quantified by Li-Cor Odyssey classic imager (LiCor Biosciences) with Image Studio software. Data are means and standard of 3 experiments. **** *P* < 0.0001, *** *P* < 0.001, * *P *= 0.014 by one-way ANONA (GraphPad 6.0). (F) HEK-293T-hACE cells (2 × 10^4^ cells/well) were seeded onto 96-well plates and pretreated with 100 µl of serial 2-fold dilutions of NH4Cl (25–1.6 mM) at 37°C for 1 h, followed by spin infection with pseudoviruses prepared in fresh media containing NH4Cl (25–1.6 mM). The plate was incubated at 37°C for 18 h before replacing with fresh media and miRFP signals were quantified at 48 h post-infection. Data are means and standard deviations of triplicates from one representative experiments of two.

To further characterize the pseudoviruses, qRT-PCR was performed and revealed comparative viral RNA copies in the 3 pseudoviruses and vector only (Figure 3(A)). Western blot analysis of pseudovirus particles and vector only revealed comparable amounts of HIV-1 p24, p31, p40, p49, p51, p55 and p65 when probed with HIV-1 positive sera and S proteins when probed with rabbit sera and a human mAb to SARS-CoV-1 S protein (Figures 3(B) and 3(C)). With the AAAR cleavage site mutation only S protein was found in AAAR, whereas S, S1 and S2 proteins were detected in Str and D614G. Compared with D614G, Str had comparable amounts of S2 protein, slightly increased S protein and greatly reduced S1 protein. Using the ratio of the intensity of S2 or S1 protein to that of total S protein to calculate the % cleavage, we found Str and D614G had comparable % cleavage based on S2 but greatly reduced % cleavage based on S1 (11% vs. 51%), suggesting increased S1 shedding in Str pseudovirus (Figure 3(D)). Consistent with this, Str and D614G had a similar ratio of the intensity of S2 to p24 intensity but greatly reduced ratio of S1 to p24, suggesting lower S1 density on Str pseudovirus particles compared with D614G particles (Figure 3(E)). We further examined the mechanism of entry by pretreating cells with different concentrations of NH4Cl. Similar to the effect on the control VSV-G pseudovirus, NH4Cl inhibited the infectivity of the 3 pseudoviruses in a dose dependent manner (IC_50_: 4.94–5.89 mM), suggesting they enter cells through receptor-mediated endocytosis (Figure 3(F)). Notably, repeated measurement at 72 h revealed a similar pattern of inhibition (IC_50_: 6.19–6.68 mM) (Figure S2).

### Neutralization test based on SARS-CoV-2 pseudoviruses with miRFP reporter

**Figure 4.**
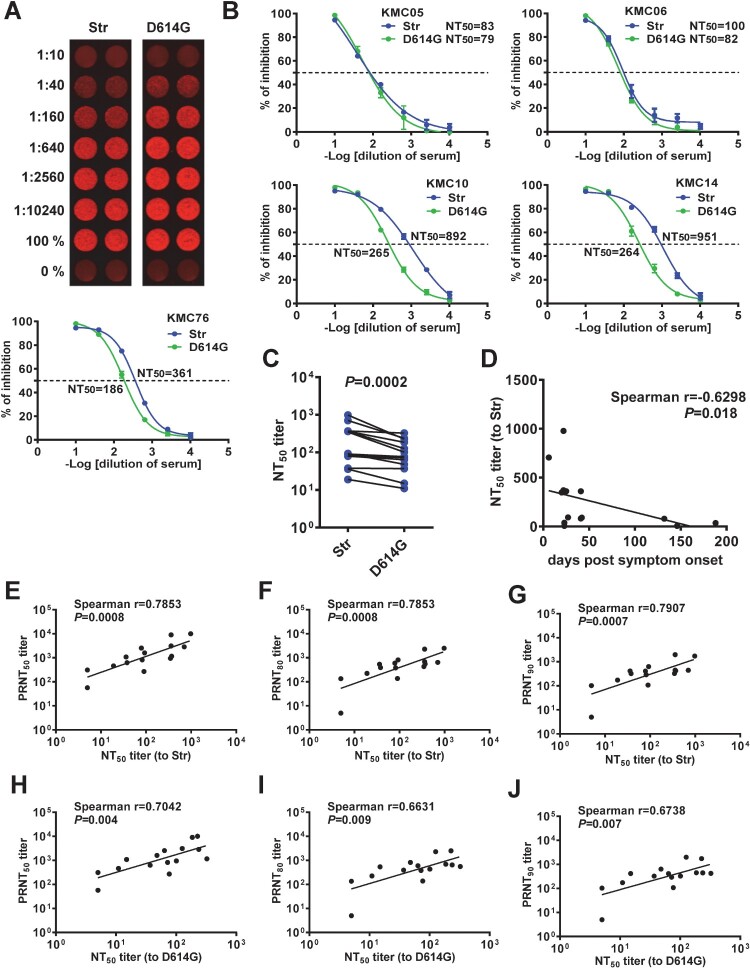
Neutralization test based on SARS-CoV-2 pseudoviruses with miRFP reporter. (A,B) miRFP signals (top) and neutralization curves and NT_50_ titers (bottom) to pseudoviruses Str and D614G at 72 h post-infection in HEK-293T-hACE2 cells of a convalescent-phase serum sample from a confirmed COVID-19 case (A) and neutralization curves and NT_50_ titers of other 4 confirmed COVID-19 cases (B). Data are means and standard deviations of duplicates from one representative experiment of two. (C) Comparison between NT_50_ titers to pseudoviruses Str and D614G for 15 confirmed COVID-19 cases. The two-tailed Wilcoxon rank signed test (GraphPad 6.0). (D) Relationship between NT_50_ titers to pseudovirus Str and sampling days. (E-J) Relationship between NT_50_ titers to pseudoviruses Str and PRNT_50_ (E), PRNT_80_ (F) or PRNT_90_ (G) titers to SARS-CoV-2 USA-WA-1 strain (containing D614) and relationship between NT_50_ titers to pseudoviruses D614G and PRNT_50_ (H), PRNT_80_ (I) or PRNT_90_ (J) titers to SARS-CoV-2 USA-WA-1 strain. The two-tailed Spearman correlation test (GraphPad 6.0).

We next employed pseudoviruses Str and D614G to perform neutralization test for confirmed COVID-19 cases. Based on the miRFP signals at 72 h, a neutralization curve was generated with NT_50_ titers of 361 (to Str) and 186 (to D614G) for one case (Figure 4(A)). Neutralization curves of other 4 cases are shown in Figure 4(B); similar curves and comparable NT_50_ titers were obtained by measurements between 48 and 96 h (Figure S3). Analysis of 15 samples revealed that NT_50_ titers to Str were higher than those to D614G (Figures 4(C) and S4, Table S1) and NT_50_ titers decreased as sampling days increased (Figure 4(D)). Comparing the NT_50_ titers to pseudovirus Str (containing D614) and PRNT_50_ titers to SARS-CoV-2 USA-WA-1 strain (also containing D614) revealed a linear relationship (spearman correlation coefficient *r* = 0.7853, *P* = 0.0008) (Figure 4(E), Table S1); similarly, a linear relationship was found when comparing with PRNT_80_ or PRNT_90_ titers (Figures 4(F) and 4(G)). Interestingly, a linear relationship was also observed when comparing the NT_50_ titers to pseudovirus D614G and PRNT_50_, PRNT_80_ or PRNT_90_ titers to USA-WA-1 strain, though not as strong as those to pseudovirus Str (Figure 4(H,J)).

We further compared NT_50_ titers and IgG binding antibodies to RBD, S1 and N proteins. NT_50_ titers to pseudovirus Str correlated with IgG binding to RBD and S1 but not with IgG binding to N protein (Figures S5(A)-S5(C)); the correlations between pseudovirus NT_50_ titers and IgG binding were not as strong as those between pseudovirus NT_50_ titers and PRNT titers, highlighting the relevance of measuring neutralizing antibodies. A similar trend of correlations between NT_50_ titers to pseudovirus D614G and IgG binding to RBD and S1 but not to N protein was also observed (Figures S5(D)-S5(F)), though not as strong as those between NT_50_ titers to pseudovirus Str and IgG binding.

## Discussion

Compared with conventional PRNT, our neutralization test based on pseudovirus with miRFP reporter can be performed in a BSL2 laboratory and requires fewer plates (one 96-well plate for 6 samples in duplicates vs. eighteen 6-well plates for PRNT), less time (48–72 h vs. 3–4 days for PRNT), less sample volume (20 µL vs. 100 µL for PRNT in duplicates, starting from 1:10 serum dilution), and is expandable to 384-well format. Compared with pseudovirus neutralization tests using luciferase reporter, our pseudovirus employed miRFP reporter which can be quantified by one step of direct imaging without multiple and laborious steps (5 min scan vs. 5 steps of ∼60 min for a 96-well plate). Moreover, the same plate can be quantified multiple times for kinetic study without generating numerous replicates. Compared with GFP reporter, miRFP has minimal autofluorescence and can be quantified by two methods: direct imaging first followed by flow cytometry using the same cells to determine percentage of positive cells, which showed a good correlation with signals from direct imaging (Figure S6). A surrogate neutralization test based on antibody-mediated blockage of ACE2-RBD interaction was recently reported as a promising high-throughput assay [42]. However, it can only detect the RBD neutralizing antibodies rather than non-RBD neutralizing antibodies identified recently [43–45]. In this regard, our pseudovirus neutralization test is more comprehensive. The positive correlations between the PRNT_50_, PRNT_80_ or PRNT_90_ titers to live virus and NT_50_ titers to pseudoviruses Str (D614) or D614G support the robustness of the assay. Together, these features suggest our pseudovirus with miRFP reporter is a simple, practical, and cost-effective tool for high-throughput neutralization. The pseudovirus can also be used to study the kinetics and mechanisms of entry, screen for entry or fusion inhibitors, and have applications in exploiting immunes responses, pathogenesis, predictors of protection, convalescent-plasma therapy, vaccine efficacy and herb immunity to SARS-CoV-2 including new variants. Given the continuous surge of multiple variants of public health concerns [11,29–31], our method can be used to quickly generate different variant pseudoviruses containing mutations in the S protein as exemplified by our D614G pseudovirus and evaluate the sensitivity to neutralization by sera from individuals after natural infection or immunization with different vaccines.

Lentivirus and VSV are known to bud at the plasma membrane; this may explain the observations of higher yield of VSV pseudovirus than SARS-CoV-2 pseudovirus as well as that of Str pseudovirus, which lacks the ER-Golgi retention signal, than S pseudovirus (Figure S1(A)). By testing with different plasmid/vector ratios and infection methods, we found a low ratio (0.5/2 µg) can generate highest yield of pseudovirus and spin infection in the absence of polybrene is very efficient (Figure 1(C)). Consistent with a previous report of pseudoviruses with tags at both N- and C-termini of S protein, our pseudovirus (without any tags in S protein) D614G had comparable viral RNA copies, less shedding and increased S1 protein density than pseudovirus Str; this may account for its higher infectivity [11,16] (Figure 3). The observation of higher infectivity of pseudoviruses AAAR than RRAR was in agreement with a recent report of fusion knock out mutant with SRAS mutations but not with other reports of TIL mutations, suggesting the effects of different mutations introduced to the fusion cleavage motif RRAR [6,9,10,16].

There are several limitations. First, we employed samples from confirmed COVID-19 cases to demonstrate the feasibility of using miRFP reporter-based pseudovirus for high-throughput neutralization; future studies involving larger sample size and longer time period of follow-up are needed to verify the methodological performance. Second, despite a wide range of sampling time from 16 to 188 days post symptom onset (Table S1) was investigated in this study, the majority were between 22 and 42 days (interquartile; median: 25 days). Future studies involving early and post-convalescent-phase samples are needed to validate our neutralization test. Third, NT_50_ titers to Str (D614) were generally higher than those to D614G (Figure 4(C)); this is agreement with a recent study but not with another reporting higher NT titers to D614G than to D614 [46,47]. Future studies involving all samples with known history of infection by D614 or D614G viruses are needed to clarify this discrepancy and provide possible explanations. Fourth, as low titers of cross-neutralization by SARS-CoV-1 sera have been reported using other pseudoviruses [6,14], it is likely that low titers of cross-neutralization will be observed by our pseudoviruses; this remains to be tested. Fifth, future development into an automated module is needed to promote this testing for large populations in clinical laboratories. Lastly, given the continuous transmission of SARS-CoV-2 in the community, the implementation of this neutralization test to evaluate vaccine efficacy would require a combination with other serological tests (such as our microsphere immunoassay) which target both S, N and/or other viral proteins to distinguish natural infection and vaccination.

In summary, we demonstrate that the pseudovirus with miRFP reporter is a conveninent, practical and robust method for high-throughput neutralization at a non-reference and BSL2 laboratory and has significant applications to our endeavours to fight against this pandemic. In light of immunization programmes with different vaccines taking place in multiple countries and the emergence of new variants of global concerns, our pseudovirus neutralization assay can be carried out to rapidly assess neutralization of these variants by sera from natural infection and various vaccinated populations in the near future.

## Supplementary Material

Figs-SARSCoV2-miRFP-EMI-FigS1-S6TableS1_editable.docx
