# Supplementary material for: A real-time and high-throughput neutralization test based on SARS-CoV-2 pseudovirus containing monomeric infrared fluorescent protein as reporter
Source: Emerg Microbes Infect. 2021 May 18;10(1):894–904. doi: 10.1080/22221751.2021.1925163 (PMC8143625; doi:10.1080/22221751.2021.1925163)
Supplement: Figs-SARSCoV2-miRFP-EMI-FigS1-S6TableS1_editable.docx [file TEMI_A_1925163_SM9386.docx]

# Fig. S1


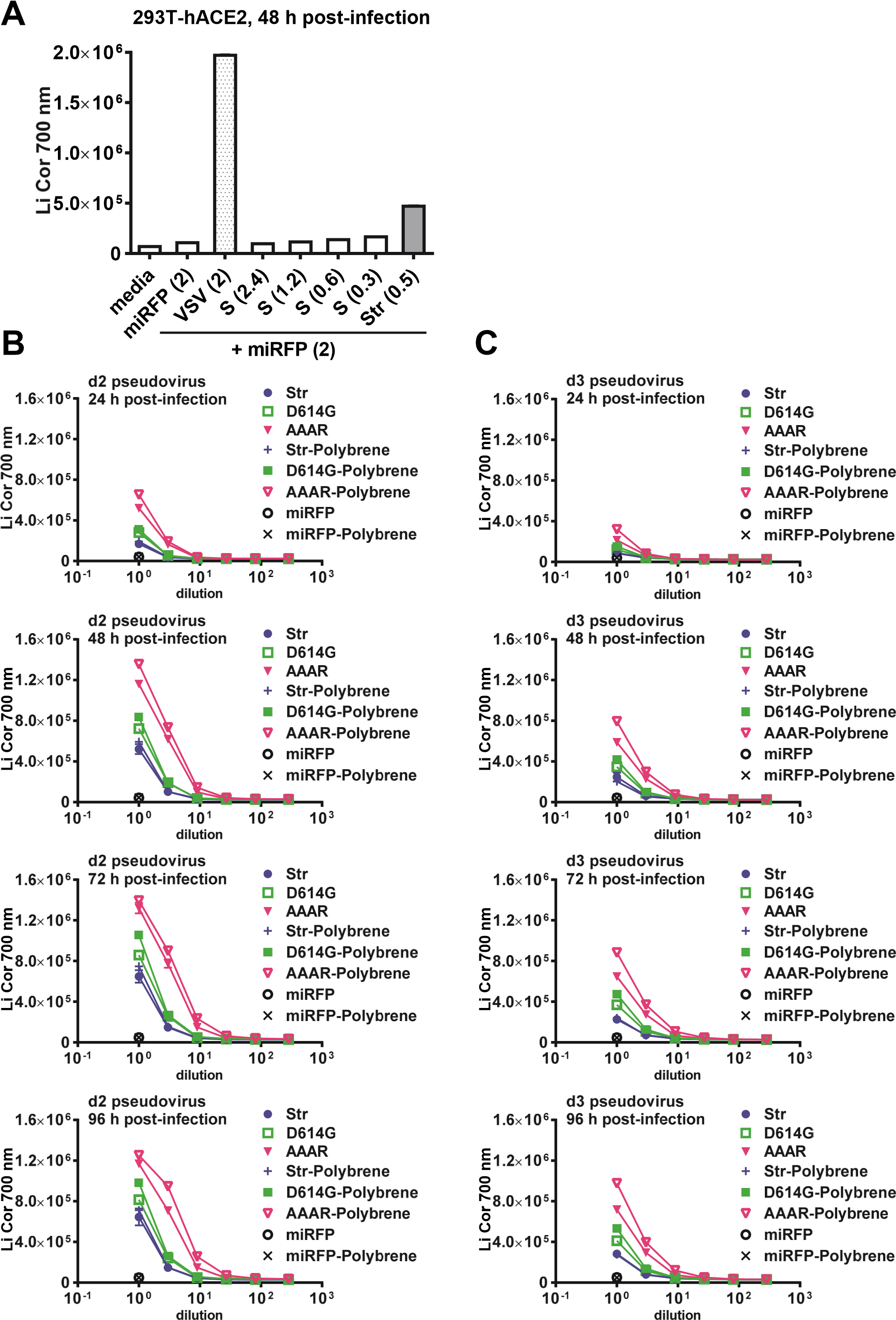


**Figure S1.** Infectivity of SARS-CoV-2 pseudoviruses collected on different days and effect of polybrene. (A) Yields of SARS-CoV-2 pseudovirus S determined in HEK-293T-hACE2 cells at 48 h post-infection comparing different ratios of S plasmid to vector (2.4, 1.2, 0.6, 0.3 µg/2 µg) during transfection, pseudovirus Str (0.5 µg/2 µg), media, miRFP vector only and VSV pseudovirus (2 µg/2 µg) as positive control. (B-C) Pseudoviruses (Str, D614G, AAAR) and miRPF vector only collected from day 2 (B) and day 3 (C) culture supernatants after transfection in 293T cells were serially 3-fold diluted and used to infect HEK-293ThACE2 cells by spin infection with or without pre-treatment of cells with polybrene; miRFP signals were quantified at 24 h post-infection. Data are means and standard deviations of duplicates from one representative experiment of two.

# Fig. S2

**A**

**0**

**5**

**10**

**15**

**20**

**25**

**0**

**20**

**40**

**60**

**80**

**100**

**NH**

**4**

**Cl (mM)**

**% of control**

**Str 5.89**

**D614G 5.52**

**AAAR 4.94**

**VSV 11.54**

**IC**

**50**

**(mM)**

**0**

**5**

**10**

**15**

**20**

**25**

**0**

**20**

**40**

**60**

**80**

**100**

**NH**

**4**

**Cl (mM)**

**% of control**

**Str 6.65**

**D614G 6.68**

**AAAR 6.19**

**VSV 13.09**

**IC**

**50**

**(mM)**

**B**

**Figure S2.** Treatment with NH4Cl inhibited the infectivity of SARS-CoV-2 and VSV pseudoviruses.

(A-B) HEK-293T-hACE cells (2 x 10^4^ cells/well) were seeded onto 96-well plates and pretreated with 100 µl of serial 2-fold dilutions of NH4Cl (25 to 1.6 mM) at 37°C for 1 h, followed by spin infection with pseudoviruses prepared in fresh media containing NH4Cl (25 to 1.6 mM). The plate was incubated at 37°C for 18 h before replacing with fresh media and miRFP signals were quantified at 48 h (A) and 72 h (B) post-infection. Data are means and standard deviations of triplicates from one representative experiments of two.

# Fig. S3


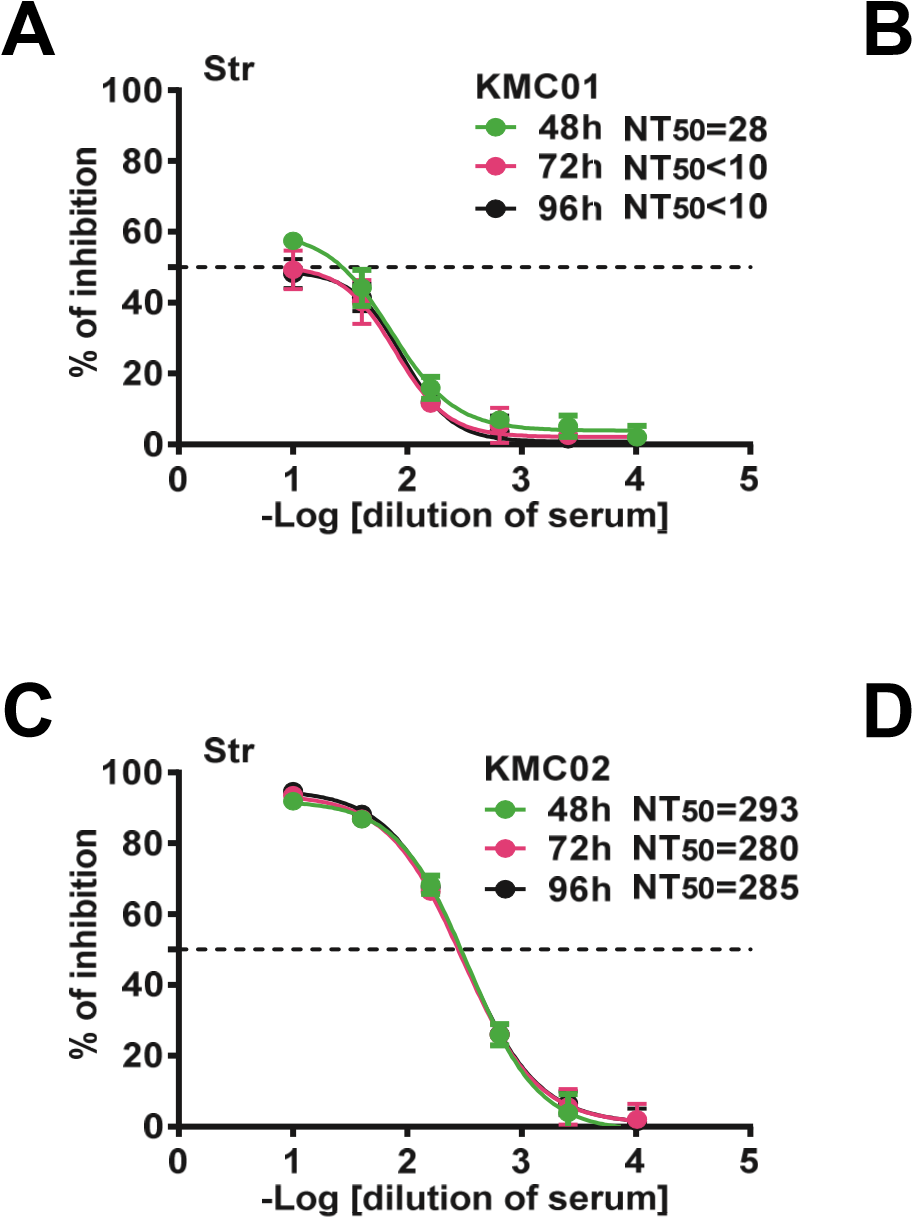

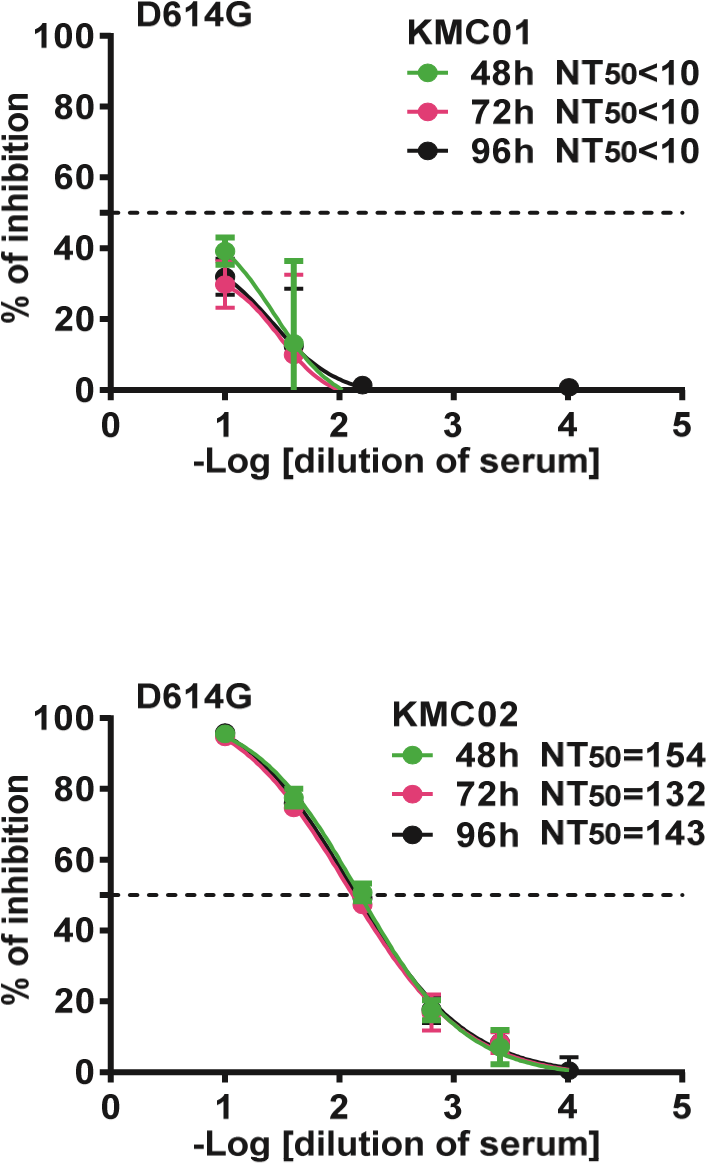


**Figure S3.** Real-time monitoring of neutralization by SARS-CoV-2 pseudoviruses with miRFP reporter. (A-D) Neutralization curve and NT_50_ titers to pseudoviruses Str and D614G at different time points (48 h, 72 h and 96 h) post-infection in HEK-293T-hACE2 cells of two confirmed COVID-19 cases KMC01 (A,B) and KMC02 (C,D). Data are means and standard deviations of duplicates from one representative experiment of two.

# Fig. S4


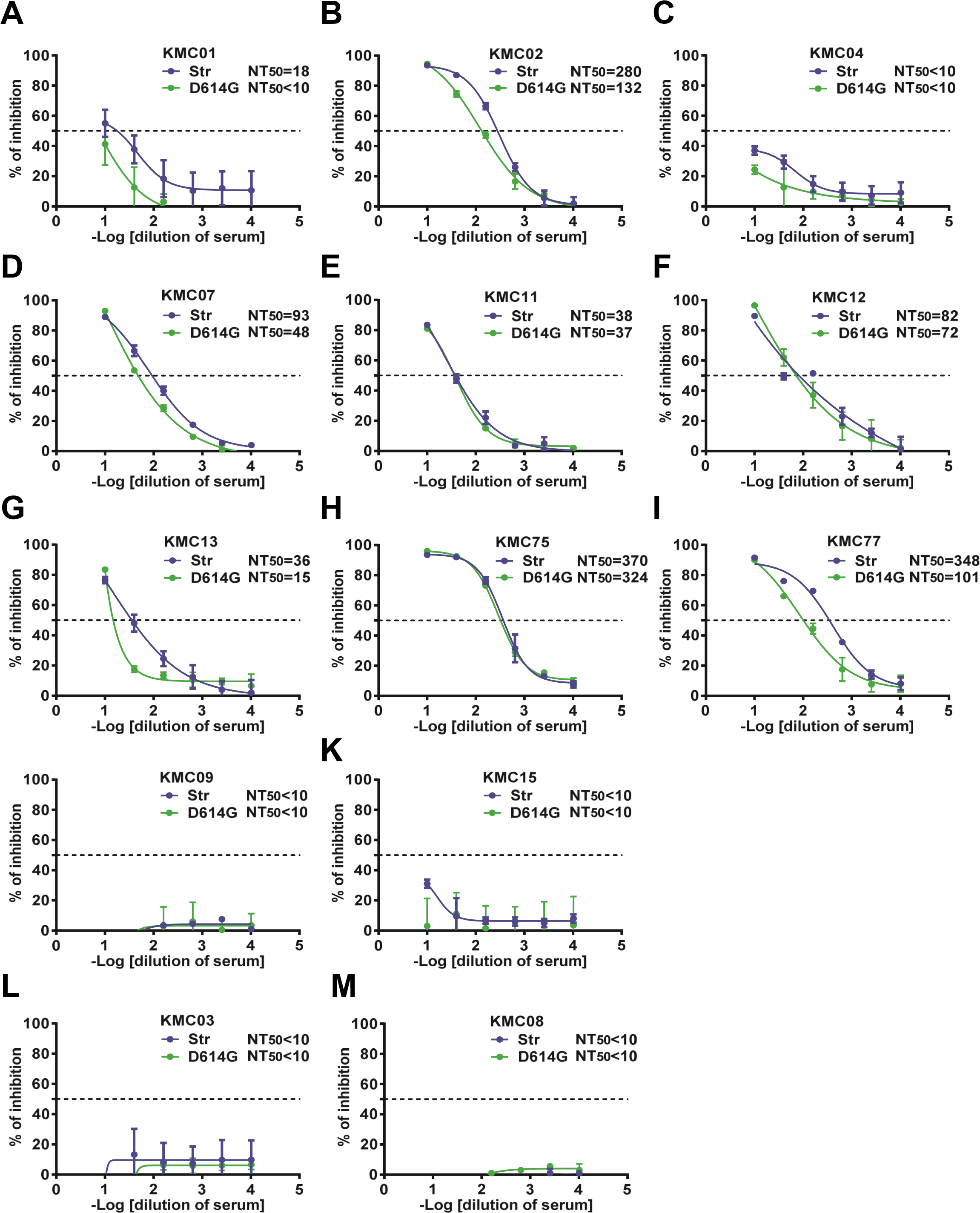
**J**

**Figure S4**. Neutralization test based on SARS-CoV-2 pseudoviruses. (A-M) Neutralization curves and NT_50_ titers to pseudoviruses Str and D614G at 72 h post-infection in HEK-293T-hACE2 cells of 11 confirmed COVID-19 cases (A-K) and 2 negative controls (L,M). Data are means and standard deviations of duplicates from one representative experiment of two.

# Fig. S5


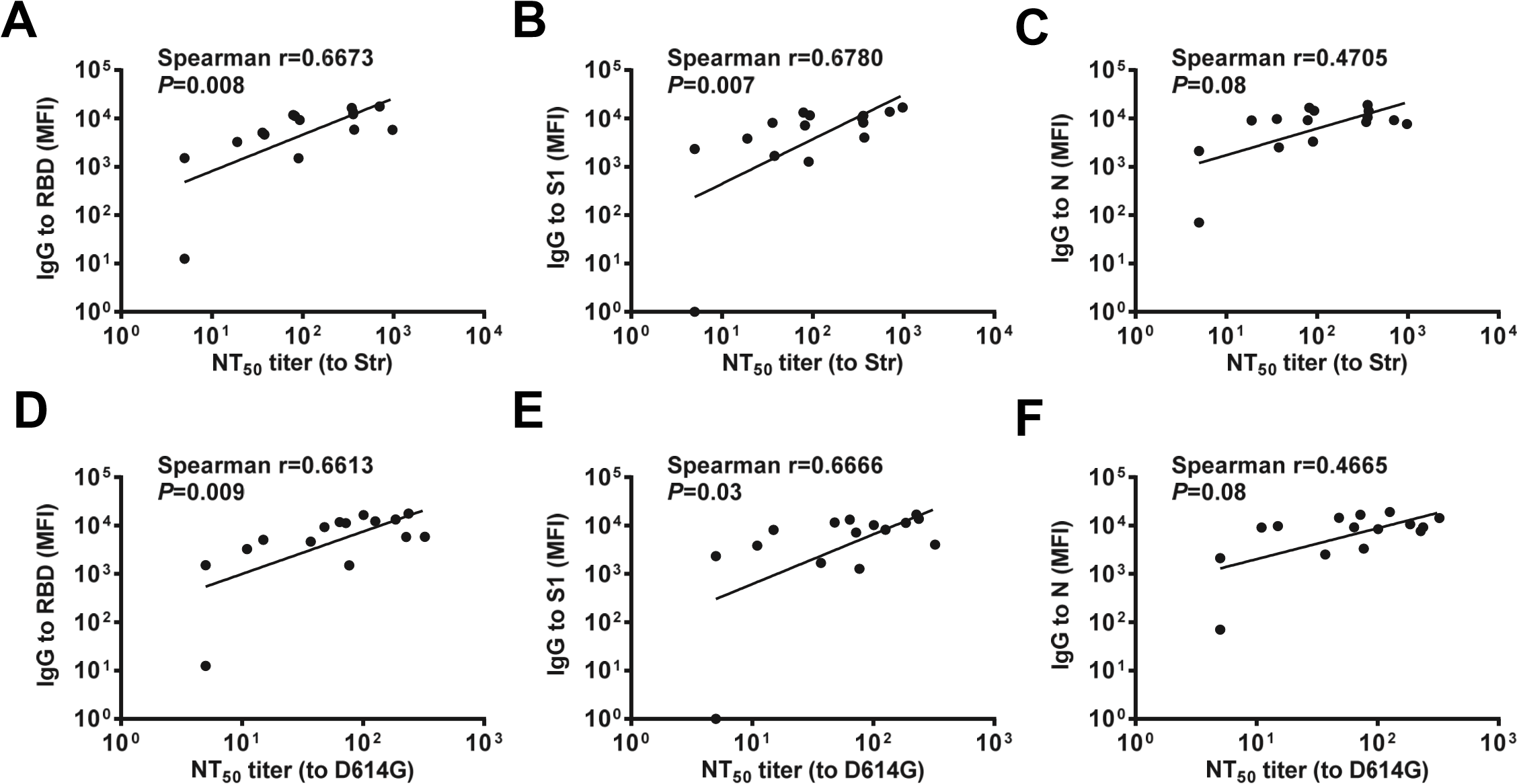


**Figure S5.** Comparison between NT_50_ titers to pseudoviruses and IgG binding to different SARS-CoV-2 proteins. (A-C) Relationship between NT_50_ titers to pseudovirus Str and IgG binding to RBD (A), S1 (B) or N (C) protein. (D-F) Relationship between NT_50_ titers to pseudovirus D614G and IgG binding to RBD (D), S1 (E) or N (F) protein. MFI, median immunofluorescence intensity in microsphere immunoassay. Data are means from two experiment each in duplicates. The two-tailed Spearman correlation test (Graphpad Prism 6).

**Fig. S6**

# A B


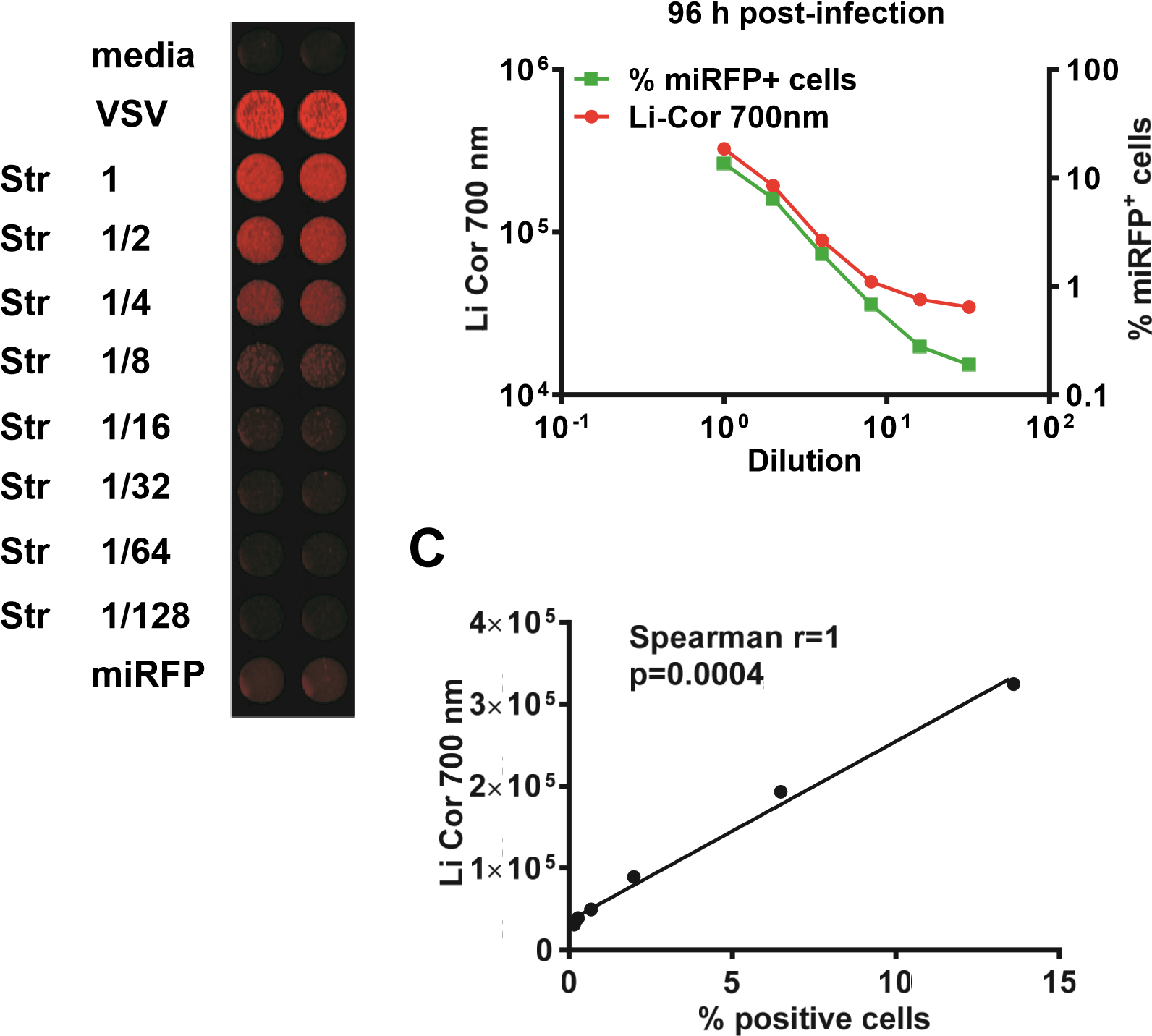


**Figure S6.** Comparison of infectivity of VSV pseudovirus with miRFP reporter quantified by direct imaging and flow cytometry. (A-C) HEK-293T-hACE cells (2 x 10^4^ cells/well) were seeded onto 96-well plates and infected with serial 2-fold dilutions of VSV pseudovirus. At 48 h post-infection, the miRFP signals were detected by Li-Cor Odyssey CLx near-infrared fluorescence imaging system (A) and quantified (B). The cells were then washed, trypsinized, fixed and counted using Attune NxT flow cytometer to determine the percentage of positive cells (B). The correlation with miRFP quantification was determined by the two-tailed Spearman correlation test (C) (GraphPad Prism 6.0).

| ID | Sampling time days PSO^b^ | Pseudovirus NT test^a^ | | | PRNT to USA-WA-1 strain^a^ | | |
| --- | --- | --- | --- | --- | --- | --- | --- |
|  |  | NT_50_ to Str (D614) | NT_50_ to D614G | PRNT_50_ | | PRNT_80_ | PRNT_90_ |
| KMC01 | N/A | 19 | 11 | 464 | | 225 | 173 |
| KMC02 | 41 | 361 | 126 | 3116 | | 2298 | 1992 |
| KMC04 | 146 | <10 | <10 | 312 | | 135 | 102 |
| KMC05 | 132 | 79 | 64 | 2545 | | 597 | 412 |
| KMC06 | N/A (42^c^) | 90 | 77 | 269 | | 138 | 107 |
| KMC07 | 27 | 93 | 48 | 1601 | | 818 | 636 |
| KMC09 | 23 | <10 | <10 | 57 | | <20 | <20 |
| KMC10 | 16 | 706 | 238 | 2850 | | 644 | 443 |
| KMC11 | 23 | 38 | 37 | 627 | | 393 | 323 |
| KMC12 | 41 | 82 | 72 | 822 | | 383 | 292 |
| KMC13 | 188 | 36 | 15 | 1081 | | 538 | 416 |
| KMC14 | 22 | 978 | 227 | 10039 | | 2500 | 1737 |
| KMC15 | 20 | <10 | <10 | 48 | | 28 | <20 |
| KMC75 | 24 | 370 | 324 | 1158 | | 553 | 424 |
| KMC76 | 25 | 361 | 186 | 9023 | | 694 | 450 |
| KMC77 | 20 | 348 | 101 | 945 | | 432 | 328 |
| KMC03 | N/A^d^ | <10 | <10 | <20 | | <20 | <20 |
| KMC08 | N/A^d^ | <10 | <10 | 28 | | <20 | <20 |

**Table S1.** Sampling time and SARS-CoV2 pseudovirus and virus neutralization tests ^a^NT, neutralization; PRNT, plaque reduction neutralization test. Data are mean and standard deviations of triplicates from two to three experiments. ^b^PSO, post symptom onset; N/A, not applicable due to asymptomatic infection ^c^days post initial COVID-19 RT-PCR positive ^d^COVID-19 RT-PCR negative
